# Pain and Dyspnea During Acute Exacerbations of Chronic Obstructive Pulmonary Disease: Documentation Audit 2019–2020

**DOI:** 10.3390/jcm14010252

**Published:** 2025-01-03

**Authors:** Stephanie Y. Clarke, Marie T. Williams, Kylie N. Johnston, Annemarie L. Lee

**Affiliations:** 1 Department of Physiotherapy, School of Primary and Allied Health Care, Faculty of Medicine, Nursing and Health Sciences, Monash University, Melbourne, VIC 3199, Australia; stephanie.clarke@monash.edu; 2 Physiotherapy Department, Eastern Health, Melbourne, VIC 3128, Australia; 3 Implementation and Clinical Translation in Health (IMPACT), Allied Health and Human Performance, University of South Australia, Adelaide, SA 5001, Australia; marie.williams@unisa.edu.au (M.T.W.); kylie.johnston@unisa.edu.au (K.N.J.); 4 Institute for Breathing and Sleep, Heidelberg, VIC 3084, Australia

**Keywords:** COPD, exacerbation, assessment, pain, dyspnea

## Abstract

**Background/Objectives:** Patient-reported outcome measures (PROMs) assess the severity and impact of both pain and dyspnea in those with acute exacerbations of chronic obstructive pulmonary disease (COPD), but their frequency of use in clinical practice is unknown. This study aimed to determine the point prevalence of pain and dyspnea assessment in patients hospitalized with an acute exacerbation of COPD and the measurement tools applied for this purpose in clinical practice. **Methods:** Clinical notes and observation charts of patients admitted with acute exacerbations of COPD to a metropolitan hospital in 2019 and 2020 were retrospectively audited to identify the point prevalence of pain and dyspnea assessment, the PROMs applied, and their associated focal periods. **Results:** Pain and dyspnea were assessed using a PROM in 99% and 8% of cases of acute exacerbation of COPD, respectively. All PROMs used measured symptom intensity. Focal periods were rarely reported in the assessment of pain; in the dyspnea assessment, timeframes predominantly reflected the impact of exertion. **Conclusions:** At this single health service site, in people hospitalized with an acute exacerbation of COPD, pain was more frequently assessed using a PROM than dyspnea. Understanding factors influencing clinicians’ choice of assessment tools may inform future recommendations for the assessment of these symptoms in people hospitalized with exacerbations of COPD.

## 1. Introduction

An acute exacerbation of chronic obstructive pulmonary disease (AECOPD) is an episode of worsening symptoms [1], marked by an acute increase in a patient’s dyspnea, cough, and/or sputum beyond normal day-to-day variation [2], and may necessitate a change in patient’s medication regimen or admission to hospital [3]. Between 27% [4] and 47% [5] of people with COPD report an exacerbation over a 12-month period, with between 2% and 12% having frequent (2 or more) exacerbations [4,5]. While dyspnea is recognized as a hallmark feature during an AECOPD, pain is increasingly observed as a symptom in this clinical state. A recent systematic review found the prevalence of pain and dyspnea in people hospitalized with an AECOPD to be 44% and 91%, respectively [6].

Patient-reported outcome measures (PROMs) have been developed to assess the severity and impact of both pain and dyspnea. Symptoms can be reported on unidimensional scales, such as numerical rating scales (NRS) or visual analogue scales. However, unidimensional PROMs provide a single perspective about a symptom and offer little insight into its nature, associated behavioral or physiological responses, or the impact of cultural, psychological, and emotional influences on the interpretation of the symptom [7,8]. Conversely, multidimensional PROMs used to assess pain, including the Brief Pain Inventory (BPI) and McGill Pain Questionnaire (MPQ), assess constructs such as pain intensity and quality and the impact on function and degree of interference caused by this symptom [9,10,11]. Multidimensional PROMs used to assess dyspnea, such as the Multidimensional Dyspnea Profile (MDP), quantify the sensation of dyspnea across the domains of sensory-perceptual experience, affective distress, and/or symptom impact or burden [12]. In patients admitted to the hospital with an AECOPD, a mix of PROMs have been used to assess pain and dyspnea, with the focal period commonly defined by the scale in use [6].

In clinical practice, it is unclear to what extent symptoms of pain and dyspnea are assessed in patients when they are admitted to the hospital with an AECOPD and which PROMs and focal periods are used. Gaining insight into current assessment practice for pain and dyspnea in people during an AECOPD may inform future recommendations for symptom evaluation in this clinical state and reveal key avenues for treatment.

This study aimed to investigate how frequently pain and dyspnea were assessed in patients hospitalized with an AECOPD during a 2-year window (2019–2020) and to identify the PROMs and focal periods used to assess these symptoms in clinical practice.

## 2. Materials and Methods

Ethics approval was obtained from Eastern Health Human Research Ethics Committee (LR19-108-72254) and Monash University Human Research Ethics Committee (22962). Eastern Health is a metropolitan health service across eastern metropolitan Melbourne with a catchment area of 2816 square kilometres. This network includes three acute hospitals, one of which is the Angliss Hospital. This is a 155-bed hospital servicing outer eastern Melbourne with two wards and an intensive care unit comprising the acute inpatient wards, which may be allocated to patients diagnosed with an AECOPD.

### 2.1. Participants [Cases]

Patient records [cases] eligible for inclusion in this audit were people admitted to Angliss Hospital with a primary diagnosis of “exacerbation of COPD” in each calendar year of 2019 and 2020 (1 January to 31 December inclusive). Following ethics approval, one investigator (S.Y.C) submitted a request to the Decision Support Department of Eastern Health for a report collating unit record numbers of people meeting the eligibility criteria. Using this report, clinical records and daily observation charts relevant to each patient’s admission were retrieved from Electronic Medical Records (Powerchart version 2018.01, Cerner) and Clinical Patient Folder (Infomedix) programs). Cases were screened to ascertain the ward to which they were admitted, their presenting diagnosis on admission, and their past medical history to confirm eligibility. Cases were only eligible for inclusion if they were admitted to an acute general medicine or surgical ward. Cases discharged from the emergency department or admitted to a sub-acute/rehabilitation ward were excluded, as were cases for which there was no documented diagnosis of COPD. Information contained within the Electronic Medical Records and Clinical Patient Folder was used as the data source for the audit.

### 2.2. Operationalization and Definition of Variables (Data Dictionary)

Data were prospectively planned to be retrieved for two broad domains: Case characteristics (demographics) and symptom assessment reporting (pain and dyspnea). A data dictionary was developed a priori. The dictionary was drafted by one investigator (S.Y.C) and reviewed by all members of the research team to reach a consensus on definitions. Preliminary testing of data item extraction and data dictionary definition utility was undertaken with five case records where data extraction was undertaken by one investigator (S.Y.C); results were reviewed independently by members of the research team with refinements and minor adjustments made to data item definitions within a consensus meeting. Final operational definitions for each variable are outlined in the data dictionary (Table S1).

The characteristic variables of each case were collated into five categories (Table S2). Demographic information was collected to describe the patient cohort. Admission details outlined hospitalization data during the study period. Spirometry data, where available, provided information on airflow limitation as this is a key component of the diagnosis and severity assessment of COPD [3]. Smoking history, a recognized risk factor for COPD [3], was recorded. Comorbidities were reported using the Charlson Comorbidity Index, a valid, reliable measure scoring coexisting clinical conditions that impact prognosis and mortality [13,14].

**Figure 1:**
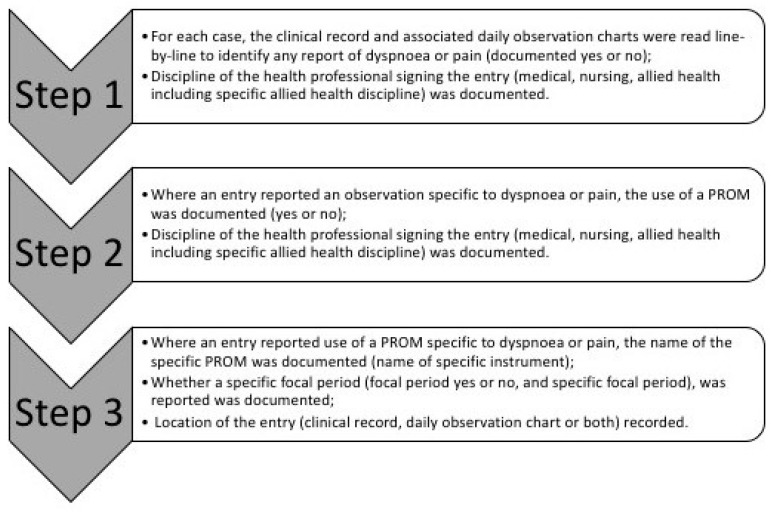
Audit process for primary data collection.

The primary data required for this audit concerned whether and how assessments of pain and dyspnea were documented within the patient’s clinical notes (medical records) or daily observation charts. For each case, one investigator (S.Y.C) extracted data as summarized in Table S3. The audit process for primary data was undertaken in a systematic, consistent process for each case record by one investigator (S.Y.C) following the steps outlined in Figure 1.

The possibility of a case having no documentation concerning pain/dyspnea, reports of one but not the other, or reports of both to varying degrees was considered. To ensure consistency of data collection, five cases were randomly re-audited for quality assurance by one investigator (S.Y.C) following extraction of all data over the study period (with 100% reproducibility and no changes made to the data obtained from the initial audit of these cases).

### 2.3. Data Management and Analysis

Data were collated into a spreadsheet (Microsoft Excel Version 16.43.1, Microsoft) and imported into Statistical Package for Social Sciences (version 27, IBM) for descriptive analysis. Primary aim: Demographic features of cases from both years were reported using mean (SD) or median (IQR) depending on data distribution. The number of cases for which the presence or absence of pain and dyspnea were documented were expressed as frequency and percentage (number and percentage of total number of included admissions). Point prevalence for the documented assessments of these symptoms using a PROM was calculated by the number of cases with documented use of a PROM as a percentage of the total number of included cases. If symptoms were assessed multiple times (using the same PROM) in the same case, documentation frequency was only counted once for the purpose of determining the prevalence of assessment. Where multiple PROMs for pain or dyspnea were reported within a single case, each specific PROM was counted within the total across all cases. The frequency of use of PROMs for pain and dyspnea was calculated by count and expressed as a percentage of the total number of cases for which there was a documented assessment of the respective symptoms. The focal periods used for the assessment of these symptoms across all cases were counted and expressed as a percentage of the total number of admissions for which a PROM was used.

## 3. Results

### 3.1. Demographic Data

**Figure 2:**
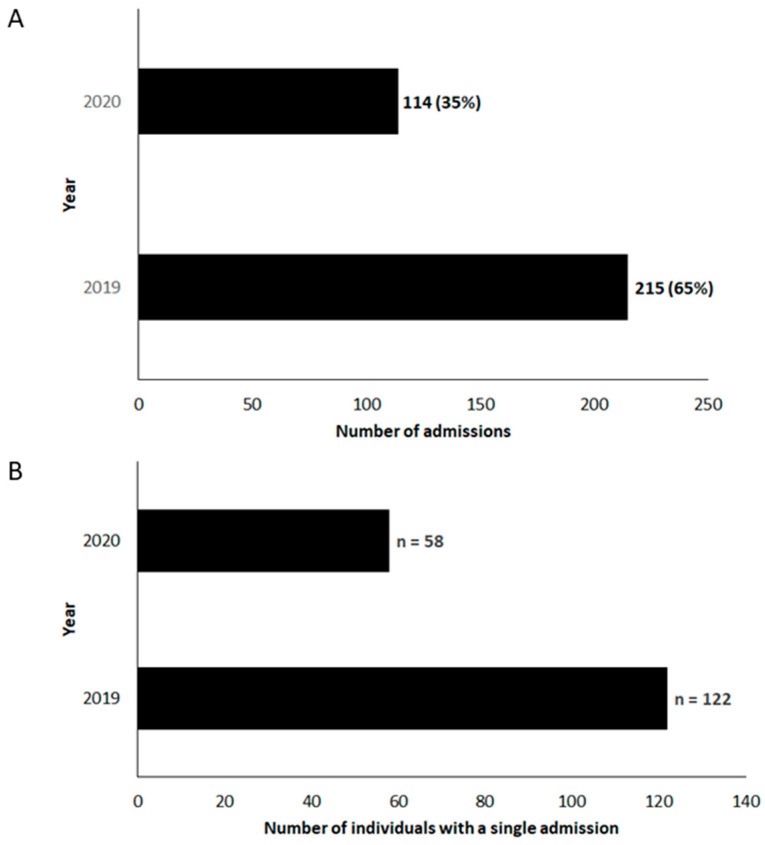
(**A**) Total number of admissions for an acute exacerbation of COPD in 2019–2020, and (**B**) total number of individuals with a single admission for an acute exacerbation of COPD in 2019–2020.

Within this single hospital site, there were 329 admissions for AECOPD from 229 individuals during 2019–2020 (Figure 2A). The number of admissions for each individual within the two-year study period ranged from one to 27. The median length of stay across all 329 cases was three days.

One hundred and eighty individuals had a single admission over a two-year period (Figure 2B). Demographic data are summarized in Table 1 for all individuals admitted with an AECOPD during the study period. The median age of individuals on their first admission within the study timeframe was 75 years, with 118 (52%) individuals being female. Spirometry data (complete or limited) were available for 131 (57%) of individuals, while it was not available for 98 (43%) of individuals.

### 3.2. Presence/Absence of Pain and Dyspnea

The number of cases for which the presence/absence or assessment of pain and dyspnea was documented and which health professionals provided the documentation are summarized in Table 2.

The absence or presence of pain and dyspnea was documented in 99% and 97% of cases, respectively. Nursing staff most frequently documented the presence or absence of pain and dyspnea and were the sole professional noting the presence or absence of pain in 50% of cases and dyspnea in 26% of cases. Allied health staff (in combination with medical and/or nursing staff) documented the presence or absence of pain in 12% of these cases (Table 2 and Table S4). In 42% of cases, the presence or absence of dyspnea was noted by allied health staff (alone or in combination with medical and/or nursing staff) (Table 2 and Table S4).

### 3.3. PROM-Based Documentation of Pain

A documented assessment of pain using a PROM was recorded in 99% of cases. Nursing staff were the sole professionals who recorded pain assessments using a PROM in 95% of cases (Table 2). All cases of pain assessment were documented in daily observation charts, with 21% of these cases also having pain assessment documented in their clinical notes. The numerical rating scale was the only PROM documented for assessments of pain (Table 3). In 96% of cases where an assessment of pain using a PROM was recorded, no focal period was specified (Table 3).

### 3.4. PROM Based Documentation of Dyspnea

While the absence or presence of dyspnea was documented in 97% of cases, only 8% of cases had a recorded assessment of dyspnea using a PROM (Table 2), which was recorded solely by physiotherapists and/or physiotherapy students (Table S4). Assessments of dyspnea were recorded only in clinical notes, with no cases of this assessment being recorded in their daily observation charts. When an assessment of dyspnea was documented, a numerical rating scale, the Borg Rating of Perceived Exertion, or the modified Borg dyspnea scale was used (Table 3). Of the cases where dyspnea was assessed using these PROMs, the focal period was reported in 88% of cases, but the actual focal period varied (Table 3), with the majority of records assessing dyspnea “at rest” and “post-ambulation”.

## 4. Discussion

In this cohort of individuals hospitalized with an AECOPD over a two-year study period, the symptom presence or absence was recorded in almost all cases for pain and dyspnea. A PROM was used for the assessment of pain in 99% of cases and for dyspnea in 8% of cases. A numerical rating scale was the only documented PROM used to quantify individuals’ pain, with focal periods rarely stated. In the small number of cases where dyspnea was documented using a PROM, numerical rating scales, Borg Rating of Perceived Exertion, or the modified Borg dyspnea scale were used, with focal periods tending to reflect the individual being at rest, during walking/exertion or post-exertion. Admissions to hospitals with an AECOPD were fewer in 2020 compared to 2019.

Since the 1990s, pain has been recommended as a vital sign, like heart rate, and ideally, measured as part of quality care [15]. The Victorian Department of Health (in operation where this study was conducted) recommends that when assessing pain in older hospitalized people, a screening question to identify the presence of pain and a self-reported pain intensity scale or a multidimensional self-report tool to quantify an individual’s pain are used [16]. Our study finding of close to 100% implementation of a unidimensional PROM to assess pain in hospitalized people with COPD confirms these recommendations are being translated, at least in part, to the local clinical setting. In over 95% of cases where pain assessment was reported using a PROM, this was recorded solely by nursing staff. The key role of nurses in the assessment and management of acute pain is well recognized [17,18]. Nurses have the most frequent engagement with patients, documenting regular assessments using daily observation charts; the high proportion of cases of pain being recognized and assessed by a nurse in this audit may reflect this practice.

Unlike pain, recommendations for assessing dyspnea during hospitalization are less well-established. This may, in part, account for the differences in PROM use between symptoms. The Australian and New Zealand Guidelines for the Management of COPD (COPD-X) recommends the use of tools, including the Modified Medical Research Council Dyspnea scale, to assess the severity of the impact of breathlessness in people with COPD [3]. The American Thoracic Society statement on dyspnea suggests that dyspnea is assessed across the multiple domains of sensory-perceptual experience, affective distress, and symptom impact [12], while the 2023 Global Initiative for Chronic Obstructive Pulmonary Disease (GOLD) report recommends a measure of dyspnea intensity using a visual analogue scale (0 to 10) in combination with other signs (respiratory rate, heart rate and oxygen saturation) for the purpose of classing exacerbation severity [19]. In addition, a mix of quality-of-life measurements validated in people with COPD, including the EuroQoL 5D, Chronic Respiratory Disease Questionnaire, and London Chest Activities of Daily Living questionnaire [3], reflect the impact of dyspnea in this population. These recommendations and options, albeit broad and not specific to individuals hospitalized with an AECOPD, contrast with the infrequent reporting of PROMs for dyspnea measurement identified in this study. This intermittent use has been previously observed. Stefan et al. [20] reported that although 90% of surveyed hospital physicians agree that awareness of dyspnea severity influences their clinical decision-making, only 2.3% of respondents used a numeric scale to assess patients’ dyspnea. Clinicians may be more likely to report increases in observed breathing work and limitations in activity or communication due to breathlessness; this was reflected by the observations of breathlessness at rest or post-exertion or ambulation in this audit study. While these observations recognize the impact of dyspnea on the individual’s presentation, they fail to quantify or evaluate the individual’s experience of this symptom. Depending on the selection, this is one of the advantages offered by the use of a PROM for dyspnea assessment in this clinical context, with the potential opportunity to provide more nuanced information that could inform treatment efficacy. The absence of a unidimensional dyspnea measure on observational charts is a possible contributor to the current audit; this omission may influence the practice of some clinicians. It is noteworthy that physiotherapists and physiotherapy students were the only professionals to report a PROM for dyspnea in this study. During an AECOPD, dyspnea may be a major factor impacting individuals’ ability to participate and progress with exercise and mobility, a focus of physiotherapy practice in COPD [3], including during acute exacerbation management [21,22,23]. Physiotherapy use of PROMs enables close monitoring of dyspnea’s severity and the impact of exertion and subsequent rest.

Less than 10% of clinical practice guidelines for COPD management refer to pain as a symptom affecting this population [24], and a systematic review evaluating the management of COPD exacerbations identified that pain is not a routinely considered outcome [25]. There is an absence of current guidance regarding how this symptom should be assessed in this patient population [26]. This may account for the lack of consensus on the optimal tools or focal period to explore this symptom in this clinical state. While three different PROMs were documented for assessing dyspnea compared to one PROM applied to quantify pain, all were unidimensional measures of symptom intensity. Even as unidimensional PROMs, the numerical rating scale is linear, while the modified Borg scale is non-linear, which makes comparisons of scores between scales challenging. The modified Borg scale (with word anchors) means that a high level of dyspnea is required to achieve a high score, which contrasts with that of the numerical rating scale. Given that the symptoms of pain and dyspnea are aversive and inform medical and physiotherapy management, it is unclear why unidimensional are favoured for use by clinicians when multidimensional tools offer detail on both the sensory and affective domains of these symptoms. It may, in part, be related to the perspective of patients, where a preference for unidimensional tools for measuring dyspnea has been identified [27]. However, multidimensional tools offer greater detail of the clinical implications of pain and dyspnea and, importantly, may provide further insight into the efficacy of specific treatment options and how they address both the sensory and affective domains of an individual. Both the MPD and the Dyspnea-12 have been proposed as suitable measures that assess symptom domains and their response to treatment [24]. The MDP has been applied in those with an AECOPD, with reports of a decline in the sensory qualities and affective responses to dyspnea during hospitalization, indicating improvement [28]. This suggests that such tools are responsive to treatment in this clinical context. The choice of unidimensional tools may be practical, but healthcare professionals have limited time to complete symptomatic assessments in the clinical setting. While focal periods can add to the context of pain and dyspnea, they can also be potentially affected by memory and recall bias. Despite pain and dyspnea affecting 44% and 91% of individuals hospitalized with exacerbations of COPD [6], respectively, it is unclear which factors drive clinicians’ practice when assessing these symptoms. It could be the intensity of the symptom, which may be in part determined by the scale of the PROM (linear versus non-linear). The decision-making behind clinicians’ selection of PROMs, the inclusion of a focal period, and its specific context when assessing pain and dyspnea in those with an AECOPD remains an area of future research. While some tools have a pre-determined focal period, which is inherent to validity and reliability, others are less specific and may be adapted for optimal clinical application.

The clinical implications of the presence and intensity of pain and dyspnea lend support to the importance of recognizing the presence of each symptom during hospitalization. In one of the largest studies to date investigating the impact of dyspnea in greater than 50,000 hospitalized patients in the United States of America, the presence of dyspnea at any intensity upon hospital admission was associated with an increased mortality risk and greater use of healthcare resources [29]. In people with AECOPD, pain is associated with poorer health status and increased fatigue, anxiety, and depression [30], while higher pain intensity is correlated with increased dyspnea, anxiety, and depression [31]. For those with an AECOPD, self-report scales identifying changes in symptoms, including dyspnea, are considered clinically useful [32]. Patient-reported outcome measures for pain and dyspnea have the potential to provide valuable information regarding symptom characteristics and their impact on individuals, which may guide treatment options. This is essential to devising recommendations for consistent use of tools and focal periods or context to assess these symptoms in this patient cohort. Furthermore, while this audit provides insight into the frequency of use of some PROMs, details of the inter-rater reliability of these tools (due to use between multiple clinicians) and temporal changes in the measurement of symptoms during hospitalization for an AECOPD may further inform overall clinical management for these individuals.

A limitation of this study is that data were retrieved from only one hospital in a metropolitan health network, which did not have a specific respiratory unit. As individuals admitted to this hospital with acute exacerbations of COPD were assessed and treated by general medicine physicians and clinicians, the findings may vary at hospitals with respiratory units, where doctors, nurses, and allied health staff may have greater exposure to this clinical condition. This may limit the generalizability of the findings as a reflection of the assessment practice of clinicians caring for those with an AECOPD. While pain is assessed routinely as part of standard observation charts completed by nursing staff at this hospital, there is no protocol associated with the assessment of either symptom during admission for AECOPD. This study excluded cases who were discharged directly from the emergency department or short-stay unit; findings cannot be extrapolated to reflect clinical practice in these areas. Patient files audited in this study were of individuals who were admitted with a primary diagnosis of exacerbation of COPD. There may be a small set of cases that were excluded due to presenting with a differing primary diagnosis but subsequently receiving treatment for an exacerbation of COPD during their admission. The years included in this audit coincided with the first year of the COVID-19 pandemic. The number of admissions is likely to have been less for AECOPD due to restrictions on public gatherings and strict social distance measures introduced to limit virus transmission with stay-at-home orders [33,34,35].

## 5. Conclusions

In this study, the presence or absence of pain and dyspnea was frequently documented by health professionals caring for individuals hospitalized with an AECOPD within a single hospital. While pain assessment using a PROM was documented in nearly all patients in this cohort, dyspnea was rarely assessed using a PROM. All documented PROMs for either symptom were unidimensional. The documentation of focal periods was sporadic in pain assessments but more prevalent in the assessment of dyspnea. Exploration via qualitative research to obtain in-depth evaluation into clinicians’ beliefs and understanding of these symptoms, factors influencing their choice of assessment tools, and interactions between pain and dyspnea may inform future recommendations for assessment of these troubling symptoms in people hospitalized with exacerbations of COPD. This exploration should include the current use of focal periods for evaluation to gain insight into how this may be standardized when possible in clinical practice to improve evaluation. This knowledge is the first step towards refining a precision medicine approach to clinical care for this population, which includes the most optimal assessment tool for pain and dyspnea and what training for clinicians may be necessary to ensure implementation within practice.

## Figures and Tables

**Table 1:** Characteristics of included individuals (*n* = 229).

	Data Available for *n*	N (%) or Median (IQR) Unless Otherwise Stated
Demographic features		
Sex *	*n* = 229	Female: 118 (51.5%)
Age on first admission during the study period (years) †	*n* = 229	75 (65.0–82.5)
Number of admissions in 2019–2020 *	*n* = 229	
1		180 (78.6%)
2		29 (12.7%)
3		13 (5.7%)
4		5 (2.2%)
5		1 (0.4%)
27		1 (0.4%)
Charlson Comorbidity Index on entry to study †	*n* = 229	5.0 (4.0–7.0)
Non-English-speaking background *	*n* = 229	4 (1.7%)
**Social situation**		
Living arrangement on first admission during the study period *	*n* = 229	
Home alone		61 (26.6%)
Home with others		146 (63.8%)
Residential care		21 (9.2%)
Unknown		1 (0.4%)
Change in living arrangement during study *	*n* = 229	3 (1.3%)
**Oxygen therapy and Smoking status**		
Home oxygen on first admission during the study period *	*n* = 229	
Yes		23 (10.0%)
No		206 (90.0%)
Change in home oxygen status during study *	*n* = 229	4 (1.7%)
Smoking status on entry to study *	*n* = 229	
Current smoker		81 (35.4%)
Ex-smoker		133 (58.1%)
Unknown		15 (6.6%)
Change in smoking status during study *	*n* = 229	6 (2.6%)
Pack year history on entry to study *	*n* = 229	
0–19 years		27 (11.8%)
20–39 years		48 (21.0%)
40–49 years		53 (23.1%)
60+ years		37 (16.2%)
Pack year history not available		64 (27.9%)
**Spirometry measurements**		
FEV_1_ % predicted †	*n* = 126	46.0 (33.0–64.0)
FEV_1_ % predicted change post-bronchodilator †	*n* = 119	6.0 (1.0–12.0)
FVC % predicted †	*n* = 126	75.0 (62.0–89.0)
FVC % predicted change post-bronchodilator †	*n* = 119	6.0 (2.0–12.0)
FEV_1_/FVC ratio †	*n* = 126	46.0 (36.75–62.25)
FEV_1_/FVC ratio % predicted †	*n* = 108	62.0 (47.25–85.0)
FEV_1_/FVC ratio post-bronchodilator †	*n* = 124	47.0 (36.0–61.0)

* values expressed as a percentage; female was the most commonly reported sex in this audit; † values expressed as median (interquartile range); FEV_1_ = forced expiratory volume in 1 s; FVC = forced vital capacity; IQR—interquartile range; N = number; *n* = sample size.

**Table 2:** Summary of documentation (presence/absence and use of PROM for assessment) for pain and dyspnea by profession.

Count of Documented Presence/Absence and Use of PROM for Assessment of Pain and Dyspnea by Profession				
	Pain (*n* = 329)		Dyspnea (*n* = 329)	
	Presence/ Absence	Use of PROM	Presence/ Absence	Use of PROM
Yes	325 (98.8%)	325 (98.8%)	318 (96.7%)	25 (7.6%)
No	4 (1.2%)	4 (1.2%)	11 (3.3%)	304 (92.4%)
Doctor only	0 (0%)	0 (0%)	8 (2.5%)	0 (0%)
Nursing staff only	162 (49.8%)	309 (95.1%)	81 (25.5%)	0 (0%)
Allied health only	0 (0%)	0 (0%)	1 (0.3%)	25 (100%)
Doctor and nursing staff	125 (38.5%)	11 (3.4%)	96 (30.2%)	0 (0%)
Nursing staff and allied health	12 (3.7%)	3 (0.9%)	42 (13.2%)	0 (0%)
Doctors, nursing staff, and allied health	26 (8.0%)	2 (0.6%)	90 (28.3%)	0 (0%)

Data are N (%)*. n* = sample size; PROM = patient reported outcome measure.

**Table 3:** Patient-reported outcome measures and focal periods used to assess pain (*n* = 325 admissions) and dyspnea (*n* = 25 admissions).

	Pain		Dyspnea	
PROM	Focal Period	N (%)	Focal Period	N (%)
NRS (0–10) ‡	Not specified	312 (96.0%)	Not specified	3 (12.0%)
	Specific time *	1 (0.3%)	Post-ambulation/exertion	7 (28.0%)
	“Post analgesia” or “post-endone” *	2 (0.6%)	At rest and post-ambulation	1 (4.0%)
	“With cough” or “on cough” *	3 (0.9%)	On ambulation, then at rest (following ambulation)	1 (4.0%)
	“When breathing in” *	1 (0.3%)		
	“In early morning” *	1 (0.3%)		
	“When breathing in and out” *	1 (0.3%)		
	Headache score post-GTN *	1 (0.3%)		
	“With breathing” and “on movement” †	1 (0.3%)		
	“Throughout movement” *	1 (0.3%)		
	“After brushing teeth in the toilet” *	1 (0.3%)		
RPE (*n* = 2, 8%)	N/A		At rest	1 (4.0%)
			Post-ambulation *	1 (4.0%)
mBorg (*n* = 9, 36%)	N/A		At rest	3 (12.0%)
			Post-ambulation/exertion	4 (16.0%)
			At rest and post-ambulation	2 (8.0%)
NRS (0–10) ‡ and mBorg (*n* = 2, 8%)	N/A		At rest and post-ambulation	1 (4.0%)
			Post-ambulation *	1 (4.0%)

Data are N (%). * documented once, otherwise not specified; † each circumstance documented once, otherwise not specified; ‡ exact metric being measured was not stated, but assumed to be intensity; reported in pain (325 admissions (100%); dyspnea (12 admissions (48%)); GTN = glyceryl trinitrate; N = number; *n* = sample size; N/A = not applicable; NRS = numerical rating scale; PROM = patient-reported outcome measure; RPE = Borg Rate of Perceived Exertion; mBorg = modified Borg dyspnea scale.

## Data Availability

The original data presented in this study are included in the article and Supplementary Material.

## Supplementary Materials

The following supporting information can be downloaded at https://www.mdpi.com/article/10.3390/jcm14010252/s1. Table S1. Data dictionary; Table S2. Variables collected for each case; Table S3. Data extracted from audit source; Table S4. Count the documented presence/absence or assessment of pain and dyspnea by specific allied health professionals.
