# Supplementary material for: Pain and Dyspnea During Acute Exacerbations of Chronic Obstructive Pulmonary Disease: Documentation Audit 2019–2020
Source: J Clin Med. 2025 Jan 3;14(1):252. doi: 10.3390/jcm14010252 (PMC11720982; doi:10.3390/jcm14010252)
Supplement: Supplementary file 1 [file jcm-14-00252-s001.zip › jcm-3353611-supplementary.pdf]

Table S1. Data dictionary

	Included	Excluded	Responses
**Administration details**			
Case number	For multiple admissions relevant to same patient (same UR), case number will be qualified by a, b, c etc.<br>Eg. Patient number 24 was admitted 7 times over the 2-year period – their case numbers will be 24a, 24b, 24c, 24d, 24e, 24f, 24g in chronological order of the admissions.		
Appears to meet criteria?	Admission to an acute ward at Angliss Hospital.<br>Documented diagnosis of COPD.	Admission to a subacute ward<br>No documented diagnosis of COPD	1 = Yes<br>2 = No
COVID restrictions?	Were COVID restrictions in place?<br>COVID restrictions are considered to be from March 20th to October 27th 2020, even though there were short periods within this time frame where the degree of restrictions varied, or were not in place.		1 = Yes<br>2 = No
**Demographic details**			
Age	Age in years at the time of admission, as indicated on the patient's BRADMA label from relevant admission notes.		
Sex	Sex as identified on the patient's BRADMA label from relevant admission notes.		1 = Male<br>2 = Female<br>3 = Prefer not to specify
NESB?	Is the patient noted to be of NESB or of different ethnic background?<br>Information sourced from clinical notes or emergency department notes.		1 = Yes<br>2 = No<br>3 = Not specified

Living arrangement	What is the patient's usual living arrangement at the first admission during the study period? Information sourced from clinical notes or emergency department notes.	1 = Home alone<br>2 = Home with others<br>3 = Residential care<br>4 = Unknown
	Did the patient's living arrangement change during the study period?	1 = Yes<br>2 = No
On home oxygen?	Is the patient documented to be on home oxygen at the first admission during the study period? Information sourced from clinical notes or emergency department notes.<br>Did the patient's home oxygen status change during the study period?	1 = Yes<br>2 = No<br><br>1 = Yes<br>2 = No
Year of admission	The year during which the relevant admission occurred. If the single admission spanned across multiple calendar years, the year of admission will be determined from the date of admission.	1 = 2019<br>2 = 2020
Length of stay	Number of days between date of admission and date of discharge.	
Spirometry?	Are spirometry values available? Availability of spirometry ascertained from reference to values in clinical notes or emergency department notes, or respiratory function tests available on CPF (clinical patient folder) either conducted by Eastern	1 = Yes<br>2 = No

	Health respiratory service or in correspondence from patient's general practitioner.
Date of spirometry	Date of the most recent spirometry values available.
FEV_1 % pred	The most recent test relevant to admission will be considered.<br>Where specific values (FEV_1, FVC, % predicted, BD change) are not available, will be marked as Not reported.<br>For consistency of reporting, values will be rounded to the nearest whole number.
FEV_1 (% pred post BD change)	The most recent test relevant to admission will be considered.<br>Where specific values (FEV_1, FVC, % predicted, BD change) are not available, will be marked as Not reported.<br>For consistency of reporting, values will be rounded to the nearest whole number.
FVC (% pred)	The most recent test relevant to admission will be considered.<br>Where specific values (FEV_1, FVC, % predicted, BD change) are not available, will be marked as Not reported.<br>For consistency of reporting, values will be rounded to the nearest whole number.

FVC (% pred post BD change)	The most recent test relevant to admission will be considered.<br>Where specific values (FEV_1, FVC, % predicted, BD change) are not available, will be marked as Not reported.<br>For consistency of reporting, values will be rounded to the nearest whole number.
FEV_1/FVC ratio	The most recent test relevant to admission will be considered.<br>Where specific values (FEV_1, FVC, % predicted, BD change) are not available, will be marked as Not reported.<br>For consistency of reporting, values will be rounded to the nearest whole number.
FEV_1/FVC ratio (% pred)	The most recent test relevant to admission will be considered.<br>Where specific values (FEV_1, FVC, % predicted, BD change) are not available, will be marked as Not reported.<br>For consistency of reporting, values will be rounded to the nearest whole number.
FEV_1/FVC ratio (post BD)	The most recent test relevant to admission will be considered.<br>Where specific values (FEV_1, FVC, % predicted, BD change) are not available, will be marked as Not reported.

	For consistency of reporting, values will be rounded to the nearest whole number.	
Smoker?	Is the patient a current smoker? Information will be sourced from clinical notes or emergency department notes.	1 = Yes<br>2 = No<br>3 = Unknown
Smoking pack year history available?	Is the patient's smoking pack year history documented? Information sourced from clinical notes, emergency department notes or respiratory function test documentation.	1 = Yes<br>2 = No
Smoking pack year history	Information sourced from clinical notes, emergency department notes or respiratory function test documentation. Where multiple conflicting sources are available, the value on respiratory function tests will be used as a first option. Where that is not available, the clinical notes will be used. Where no information is available about smoking pack year history, field will be marked with N/A.	
Charlson CI	Past medical history ascertained from clinical notes and emergency department notes. Charlson CI calculated using https://www.mdcalc.com/charlson-comorbidity-index-cci<br>Information to clarify severity of disease may be sourced from clinical notes, emergency department notes,	

	and CPF (including diagnostic tests, correspondence and previous admissions).		
**Documentation of symptoms of pain and dyspnea**			
Documented presence/absence of pain during admission?	Any reference to the presence or absence of pain, including but not limited to:<br>No pain<br>Nil pain<br>^ pain (where ^ indicates increase)<br>Reports pain<br>Denies pain		1 = Yes<br>2 = No
Who documented presence/absence of pain?	Which health professional documented the presence or absence of pain?<br>Where “allied health” or “other” is selected, the discipline/profession will be specified.		1 = Doctor<br>2 = Nursing staff<br>3 = Allied health<br>4 = Doctor and nursing staff<br>5 = Nursing staff and allied health<br>6 = Doctor and allied health<br>7 = Other
Documented assessment of pain during admission?	Any reference to an assessment of pain beyond an indication of presence/absence of pain.	Where the presence or absence of pain is not quantified further (eg. No pain, nil pain, ^ pain, reports pain, denies pain), the response will be marked as “No”	1 = Yes<br>2 = No
Where was assessment of pain documented?	Clinical notes and observation charts will be audited.	Emergency department notes are excluded from audit	1 = Clinical notes<br>2 = Observation charts<br>3 = Clinical notes and observation charts
Who documented assessment of pain?	Which health professional documented the assessment of pain.<br>Where “allied health” or “other” is selected, the discipline/profession will be specified.		1 = Doctor<br>2 = Nursing staff<br>3 = Allied health<br>4 = Doctor and nursing staff

			5 = Nursing staff and allied health<br>6 = Doctor and allied health<br>7 = Other
What measures of pain were used?	Any specific measure or scale used will be noted.		
What focal period was used to assess pain?	Any focal period specified in the assessment of pain will be recorded.	Where a specific measure or scale is used, but no focal period is documented, the response will be marked as “Not specified”	
Documented presence/absence of dyspnea during admission?	Any reference to the presence or absence of dyspnea, including but not limited to:<br>No dyspnea/breathless(ness)/SOB<br>Nil dyspnea/breathless(ness)/SOB<br>^ dyspnea/breathless(ness)/SOB (where ^ indicates increase)<br>Reports dyspnea/breathless(ness)/SOB<br>Denies dyspnea/breathless(ness)/SOB<br><br>The terms “dyspnea”, “breathless(ness)”, “shortness of breath” (SOB) “respiratory distress” will be considered to be used interchangeably.		1 = Yes<br>2 = No
Who documented presence/absence of dyspnea?	Which health professional documented the presence or absence of dyspnea.<br>Where “allied health” or “other” is selected, the discipline/profession will be specified.		1 = Doctor<br>2 = Nursing staff<br>3 = Allied health<br>4 = Doctor and nursing staff<br>5 = Nursing staff and allied health<br>6 = Doctor and allied health<br>7 = Other

Documented assessment of dyspnea during admission?	Any reference to an assessment of pain beyond an indication of presence/absence of dyspnea.<br>The terms “dyspnea”, “breathless(ness)”, “shortness of breath” (SOB) will be considered to be used interchangeably.	Where the presence or absence of dyspnea is not quantified further (eg. No dyspnea, nil breathlessness, ^ SOB, reports dyspnea, denies SOB), the response will be marked as “No”	1 = Yes<br>2 = No
Where was assessment of dyspnea documented?	Clinical notes and observation charts will be audited.	Emergency department notes are excluded from audit	1 = Clinical notes<br>2 = Observation charts<br>3 = Clinical notes and observation charts
Who documented assessment of dyspnea?	Which health professional documented the assessment of dyspnea.<br>Where “allied health” or “other” is selected, the discipline/profession will be specified.		1 = Doctor<br>2 = Nursing staff<br>3 = Allied health<br>4 = Doctor and nursing staff<br>5 = Nursing staff and allied health<br>6 = Doctor and allied health<br>7 = Other
What measures of dyspnea were used?	Any specific measure or scale used will be noted.		
What focal period was used to assessment dyspnea?	Any focal period specified in the assessment of dyspnea will be recorded.	Where a specific measure or scale is used, but no focal period is documented, the response will be marked as “Not specified”	
Extracts from clinical notes	Specific phrases from clinical notes referencing pain or dyspnea, (and who documented them) will be collected		

UR = unit record; COPD = chronic obstructive pulmonary disease; NESB = non-English speaking background; FEV_1 = forced expiratory volume in 1 second; FVC = forced vital capacity; BD = bronchodilator; % pred = % of predicted value; Charlson CI = Charlson co-morbidity index; N/A = not available; SOB = short of breath/shortness of breath

Table S2. Variables collected for each case

	Variable	Units of measure
Demographic	Age at first admission during the study period	Years
	Sex	Male/Female/Prefer not to specify
	Patient of non-English speaking background	Yes/No
	Living arrangement at first admission during the study period	Home alone/Home with others/Residential care
	Change of living arrangement during the study period	Yes/No
	Receiving home oxygen at first admission during the study period	Yes/No
	Change in home oxygen status during the study period	Yes/No
Admission details	Number of admissions during the study period	Frequency (n=)
	Year of admission	Year
	Length of stay	Days
Spirometry	Time from most recent spirometry to admission date	Days
	Forced expiratory volume in 1 second (FEV_1)	Litres (L)
	Forced vital capacity (FVC)	Litres (L)
	Change in FEV_1 post-bronchodilator (%)	%
	Change in FVC post-bronchodilator (%)	%
	FEV_1/FVC ratio	
	FEV_1/FVC ratio post-bronchodilator	
Smoking history	Smoking status at first admission within study period	Smoker/Non-smoker/Unknown
	Change in smoking status change during study period	Yes/No
	Pack year history	Years
Co-morbidity	Charlson Co-morbidity Index score (no units)	

N = Number

Table S3. Data extracted from audit source

Data	Response options	Examples
Was the presence or absence of pain/dyspnea recorded in the patient's clinical notes or observations charts?	Yes/No	*Yes*<br>"No SOB"<br>"Pt SOBOE"<br>"Nil resp distress"<br>"No chest pain"<br>"Pt feeling ^ back pain"<br>"ATSP re: anal pain"<br>"C/o body ache"<br><br>*No*<br>"No WOB"<br>"Talking in full sentences"
Was there a documented assessment of pain/dyspnea in the patient's clinical notes or observation charts?	Yes/No	*Yes*<br>"Pt complained of central chest pain 5-6/10"<br>"Nil c/o chest pain"<br>"mod Borg 2/10"
Which health professional documented the presence/absence of pain/dyspnea?	eg. Medical, nursing, allied health (eg. physiotherapist, physiotherapy student)	
Which health professional documented the assessment of pain/dyspnea?	eg. Medical, nursing, allied health (eg. physiotherapist, physiotherapy student)	
Where was the assessment of pain/dyspnea recorded?	Daily observation chart, clinical record or both	
Which measures of pain/dyspnea were applied during the patient's admission?	eg. Numerical rating scale	
What focal periods were used for each of the measures of pain and dyspnea during the patient's admission?	eg. Right now, in the last 7 days, over the last month, in the last year	

^ = increased/increase in; ATSP = asked to see patient; c/o = complaints of; mod Borg = modified Borg dyspnea scale; resp = respiratory; SOB = short(ness) of breath; SOBOE = short(ness) of breath on exertion; WOB = work of breathing

Table S4. Count of documented presence/absence or assessment of pain and dyspnea by specific allied health profession

	Pain		Dyspnea	
	Presence/absence (n=38)	Use of PROM (n=5)	Presence/absence (n=133)	Use of PROM (n=25)
Physiotherapist	22 (57.9%)	4 (80%)	53 (39.8%)	14 (56.0%)
Physiotherapy student	10 (26.3%)	1 (20%)	30 (22.6%)	7 (28.0%)
Allied health assistant	1 (2.6%)	0 (0%)	4 (3.0%)	0 (0%)
Occupational therapist	2 (5.3%)	0 (0%)	2 (1.5%)	0 (0%)
Physiotherapist and physiotherapy student	1 (2.6%)	0 (0%)	5 (3.8%)	4 (16.0%)
Physiotherapist and allied health assistant	2 (5.3%)	0 (0%)	19 (14.3%)	0 (0%)
Speech pathologist	0 (0%)	0 (0%)	1 (0.8%)	0 (0%)
Physiotherapist and social worker	0 (0%)	0 (0%)	2 (1.5%)	0 (0%)
Physiotherapist and occupational therapist	0 (0%)	0 (0%)	4 (3.0%)	0 (0%)
Physiotherapist and speech pathologist	0 (0%)	0 (0%)	1 (0.8%)	0 (0%)
Physiotherapy student and occupational therapist	0 (0%)	0 (0%)	1 (0.8%)	0 (0%)
Physiotherapy student and allied health assistant	0 (0%)	0 (0%)	2 (1.5%)	0 (0%)
Physiotherapist, physiotherapy student and allied health assistant	0 (0%)	0 (0%)	4 (3.0%)	0 (0%)
Physiotherapist, occupational therapist and allied health assistant	0 (0%)	0 (0%)	1 (0.8%)	0 (0%)

Data are N (%)

n = sample size; PROM = patient reported outcome measure
